# The Prolonged Effect of Shift Work and the Impact of Reducing the Number of Nightshifts on Arterial Stiffness—A 4-Year Follow-Up Study

**DOI:** 10.3390/jcdd10020070

**Published:** 2023-02-06

**Authors:** Marit Skogstad, Elisabeth Goffeng, Øivind Skare, Erika Zardin

**Affiliations:** National Institute of Occupational Health (STAMI), P.O. Box 5330, 0304 Oslo, Norway

**Keywords:** shift work, pulse wave velocity, occupational health, cardiovascular disease, arterial stiffness

## Abstract

Aim: To assess changes in blood pressure (BP) and arterial stiffness among 84 rotating shift and 25 dayworkers (control subjects) at two industrial plants during a 4-year follow-up, and to assess changes in outcome variables among shift workers at the two plants after a reduction in the number of night shifts during the last year of follow-up in one of the plants. Methods: We collected demographic data using a questionnaire, examined systolic and diastolic blood pressure (sBP, dBP), central systolic and diastolic aorta pressure (cSP, cDP), augmentation pressure (AP), central pulse pressure (cPP), and pulse wave velocity (PWV). We registered sleep quality. The last 4–14 months of follow-up one plant implemented a 12-week shift plan reducing the total number of night shifts and consecutive night shifts from 16.8 to 14 and from 7.2 to 4. To assess differences in change of outcomes between study groups we applied linear mixed models. Results: The dayworkers were older, more hypertensive, reported less sleep disturbance, and smoked/snuffed less than the shift workers did. The adjusted annual increase in PWV was 0.34 m/s (95%CI, 0.22, 0.46) among shift workers and 0.09 m/s (95%CI, −0.05, 0.23) in dayworkers, yielding a significant difference of change of 0.25 m/s (95%CI, 0.06, 0.43). No significant differences were found between the two groups of shift workers in any cardiovascular disease (CVD) outcome during the last year of follow-up. Conclusions: Shift work in industry is associated with arterial stiffness, reflecting an increased risk of future CVD. No significant changes in arterial stiffness were identified as a consequence of a small reduction in the number of night shifts and consecutive night shifts.

## 1. Introduction

Shift work including night shifts and long working days are widespread and increasing. Between 10–30% of the workforce in the Western world is engaged in such work [1].

Shift work includes work beyond conventional work hours. Night shift work is defined as work of three hours or more between 23:00–06:00 o’clock, or which comprises more than seven consecutive hours including midnight and five o’clock in the morning [2,3].

Shift work increases the risk of accidents, and has been associated with endocrine disorders such as overweight, metabolic syndrome and diabetes 2, increased blood pressure (BP), sleep disturbances, and also some cancers [4]. Recent meta-analysis and systematic reviews reveal associations between shift work and disease within the cardiovascular system [5,6,7]. However, in a systematic review of systematic reviews with meta-analyses, evidence that linked shift work to broadly defined cardiovascular disease (CVD) such as stroke, was rated as being very low [8].

However, it is established that the circadian system involves a central clock (the suprachiasmatic nucleus) and peripheral clocks, in blood cells, in adipose fat tissue, and even brain tissue. The circadian clock in mammals has adapted to the 24-h day and night cycle on earth. This sheds light on the impact of night shift work as the circadian clock regulates neuronal, metabolic, and hormonal functions. Thus, disturbances of the circadian system as a result of night shift work may affect metabolic and cardiovascular health [1,9].

Arterial stiffness is regarded as one of the earliest manifestations of vascular damage [10,11], and represents a cause of hypertension [12], as well as coronary heart disease, stroke and other cardiovascular events and is linked to high levels of pulse wave velocity (PWV) [13].

It is possible to measure PWV several ways, but in the present study, we use the carotid to femoral PWV (cfPWV) approach. cfPWV is considered the gold standard method for assessing large arterial stiffness with a high degree of reproducibility [10,14].

In a systematic review of shift work and early arterial stiffness Gusmao et al. selected 11 articles in which PWV indicated arterial stiffness. Most of the studies are cross-sectional or prospective studies of short duration. Three of these papers were sub-studies within the present cohort [15,16,17]. The review concludes that the presented results of the studies are conflicting and the authors suggest longitudinal studies [18]. We have followed our cohort of rotating shift workers in industry for four years. Here, we study early manifestations of cardiovascular disease (CVD) by examining the carotid artery, blood pressure and arterial stiffness, maximal oxygen consumption (VO_2max_), but also assessing glycosylated hemoglobin, lipids, inflammatory and adhesion markers [19]. In our previous 3-year study we demonstrated that rotating shift work with night shifts is associated with in an increase in arterial stiffness by the means of PWV [20]. We also found some support for increased systemic inflammation among the shift workers compared to the dayworkers [20].

The main aim of this study was to assess changes in BP and arterial stiffness and to determine if (i) PWV continues to increase among shift workers compared to dayworkers during the 4-year follow-up and (ii) if PWV differs between shift workers who had implemented fewer night shifts in their schedule as compared to those who maintained the old shift plan schedule during the last year of the follow-up.

## 2. Material and Methods

### 2.1. Study Design and Population

The present study, initially planned to last for 3 years [19], is a 6-year prospective follow-up study in industry. Of 172 eligible workers at two insulation material plants (thereafter A, B) in Eastern Norway, 94 workers agreed to participate in the study at baseline in 2018. The sample size and decision and study power calculation are described elsewhere [19]. At plant A we recruited 42 shift workers (84%), and in plant B 23 shift workers (33%), and 29 dayworkers (55%) participated in the study. Additional shift workers were recruited for a sub-study in 2020 [21], resulting in a participation rate of 62% of all eligible shift workers at plant B. At the 4-year follow-up 29 new shift workers were included in addition to three dayworkers. One shift worker started as a day worker the first year of follow-up and four shift workers the last year of follow-up. These four were consequently regarded as control subjects and accounted for in the statistical analysis. Thus, in August 2022, the eligible number of shift workers and dayworkers were 109 and 37, respectively. From further analysis we excluded eight participants with serious medical conditions [19]. Further, 29 participants were not able to take part in the examination in 2022 due to retirement, new position in another part of the country, sick leave, vacation or simply because they refused further participation. This resulted in 84 shift workers and 25 dayworkers included in the examination and data analysis in 2022. Except for two participants who initially were shift workers at plant A, dayworkers were mostly blue-collar workers with some shift work experience and were recruited at plant B [20]. The study cohort is depicted in the flow diagram. 


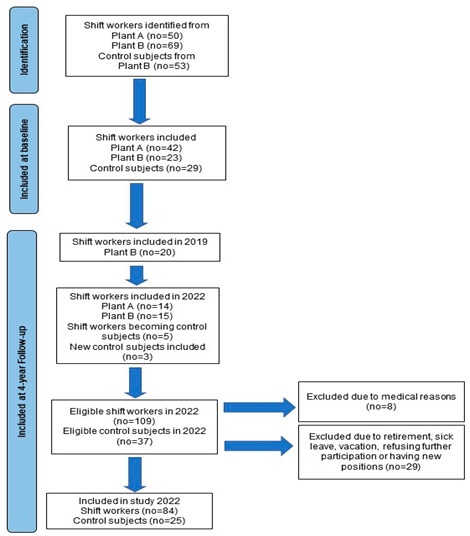


### 2.2. Demographics

We collected background data (medical history, medication, physical activity (PA), work history and smoking, snuff use or “snus” habits and history) at the 4-year follow-up using questionnaire. We measured weight in kg using a Seca 22089 balance (Seca GmbH, Hamburg, Germany). The participants provided height in cm and reported aerobic PA with a high degree of intensity in minutes per week, in an administered questionnaire developed at the Institute [16]. We calculated pack-years based on the participant’s information on no. of years as smoker and no. of daily smoked cigarettes. One pack of 20 cigarettes daily smoked during a year equals one pack-year.

### 2.3. Shift Work Exposure

**Figure 1:**
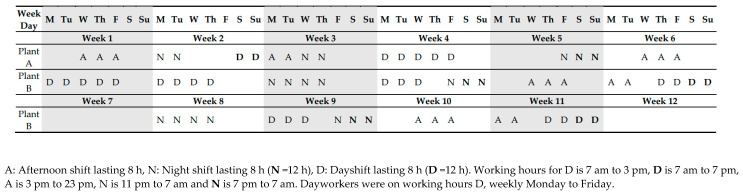
The 5-week shift plan for plant A and the 12-week shift plan for plant B in 2022.

Initially the shift workers at both plants followed a 5-week schedule. For this schedule, day, evening, and night shifts rotate in a clockwise direction. The ordinary shift plan comprises seven days shifts and seven nights shifts [19]. At plant B there was a 5-month break in shift work during 2020 [21]. This plant implemented a new shift schedule expanding on 12 weeks. The plan was introduced a median of 7 months (range: 4–14) prior to the 4-year follow-up. The number of consecutive night shifts were reduced as well as the total number of night shifts. Thus, the total number of night shifts no longer total 16.8, but 14 nights during a 12-week period and the number of consecutive nightshifts was reduced from 7.2 to 4, Figure 1.

### 2.4. Brachial Blood Pressure and Resting Heart Rate

We measured blood pressure (BP) and resting heart rate (RHR) by the means of BpTRU^®^ (Bp TRU medical devices, Coquitlam, BC, Canada). Following a five-minute rest, we performed measurements on the left arm three times in intervals of one minute while the participant was sitting. We applied the mean of three measurements of the systolic (sBP) and the diastolic pressure (dBP), and the mean of the final three measurements of resting heart rates (RHR) in the statistical analysis. The measurements were performed right after summer vacation mostly between 8 o’clock in the morning and 4 o’clock in the afternoon. We also performed measurements before evening and night shifts to exclude effects on outcome variables due to night shifts.

### 2.5. Arterial Stiffness

We assessed augmentation pressure (AP), central pulse pressure (cPP), central systolic aorta pressure (cSP), central diastolic aorta pressure (cDP), and pulse wave velocity (PWV) by the means of SphygmoCor XCEL^®^ (AtCor Medical Pty Ltd., Sydney, Australia). PWV is the speed in m/s of arterial pressure waves moving along the aorta and large arteries from the carotid to the femoral artery, cfPWV. The instrument is calibrated annually, and we carried out the measurements according to the recommendations from the manufacturer (www.atcormedical.com, accessed on 30 April 2012).

### 2.6. Sleep Measure

We used the Bergen Insomnia Scale (BIS) [22] to identify the sleep patterns of the participants in 2022. The questionnaire includes six items assessing difficulty to initiate and maintain sleep. We calculated a sum score by adding up the score for each question in the questionnaire. A higher total score indicates more reduced sleep quality.

### 2.7. Statistical Analysis

We expressed data as mean (±SD). Visual inspection of residuals Q-Q plots confirmed that all outcome variables were approximately normally distributed (plots not shown). Unadjusted differences between the shift workers and dayworkers were calculated using the independent samples t test. Linear correlations were determined using Pearson’s correlation coefficient (ρ). Linear mixed models were applied to determine whether the group of shift workers differed over the four years of follow-up from the dayworkers (control subjects) in terms of annual change in health outcomes. For this purpose, we added the two covariates total number of years since baseline and number of years of shift work since baseline as fixed effects. This approach enabled us to take into account that shift work status had changed for four participants during the last year of follow-up. Total years and years of shift work were considered continuous variables and we assumed a linear change in health outcome with year. In order to investigate the possible health effects of a reduction in number of night shifts, we also did a linear mixed analysis comparing workers with different shift-schedules at the two plants during the 4-years follow-up. This included an extra continuous variable, total number of years with new reduced shift schedule, that enabled us to estimate the difference between shift schedules in terms of annual change of health outcome. To further investigate the possible health effects of a reduction in number of night shifts, we also did a linear mixed analysis comparing shift workers at the two plants during the final year of the follow-up (at the 3-year and the 4-year follow-up). This included an interaction term between plant and time to estimate the difference between plants in the change of health outcome. All mixed model analyses were adjusted for pack-years, being a daily smoker, age, sex, and physical activity (number of minutes per week of high intensity activity) [23]. In addition, for the first two mixed model analysis, we also adjusted for a five-month break that occurred in one of the plants. The t test and the Pearson test were done using SPSS (v 28 IBM SPSS, Armonk, NY, USA). The mixed model analysis was carried out in Stata (v.17.0, StataCorp, College Station, TX, USA). Correction for multiple testing using the Benjamini-Hochberg correction was done in R.

## 3. Results

### 3.1. Demographic Characteristics in 2022

The dayworkers, compared to the shift workers, were somewhat older, more hypertensive and to a higher degree on antihypertensive medication, but smoked and used “snus” less than the shift workers. The shift workers reported more sleep problems as compared to dayworkers, Table 1.

### 3.2. Cardiovascular Outcomes over the 4-Year Follow-Up

**Figure 2:**
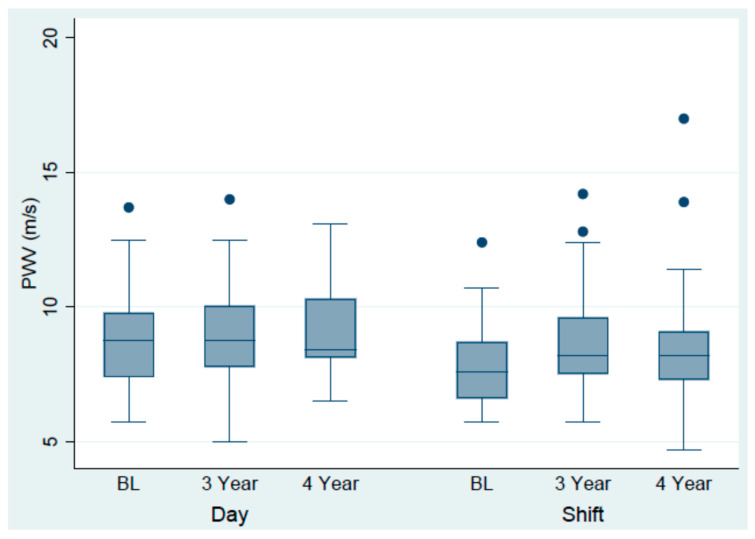
The change in PWV over the 4-year follow-up among day and shift workers.

The annual increase in measurement of the arterial stiffness measures PWV was 0.34 m/s (95% CI, 0.22, 0.46) among shift workers as compared to that of the dayworkers of 0.09 m/s (95% CI, −0.05, 0.23). This yielded a significant difference in change of 0.25 m/s (95% CI, 0.06, 0.43) in favour of the shift workers. No other differences between shift vs. dayworkers for the other outcome variables were registered, Table 2, Figure 2.

### 3.3. Cardiovascular Outcomes and Shift Work Schedules 

Over the last year of follow-up, there were no significant differences in changes of outcomes comparing shift workers at plant B (new 12-week shift schedule) with those at plant A (old 5-week shift schedule). The difference in change between plant B and A in PWV was 0.06 m/s (95% CI, −0.43, 0.55). The results for the other variables are shown in the Supplementary Table S1. Over the 4-year follow-up, we found no difference in selected variables in annual change between shift workers in the new 12-week shift schedule when this was compared to those in the old 5-shift schedule, Supplementary Table S2.

### 3.4. Correlations

At the 4-year follow-up, all blood pressure measurements were highly correlated with each other and with arterial stiffness variables, as well as with BMI, age, and pack-years (results not shown). Sleep score correlated positively with number of years of “snus” use, ρ = 0.216, *p* = 0.027. PWV correlated positively with the total number of years of shiftwork as well as with age, BMI, pack-years, BP and RHR (results not shown).

## 4. Discussion

In this 4-year follow-up study of shift workers and dayworkers in industry, we found an increase in PWV indicating a possible augmentation of arterial stiffness among the shift workers. There were no differences in increase in PWV during the last year of follow-up when comparing the shift workers with a reduced number of total night shifts and consecutive night shifts with those who remained in the original 5-shift schedule.

Higher blood pressure affects shift workers [24]. In the present study the shift workers’ sBP and dBP tended to increase during the 4-year follow-up, but the results were comparable to that of the dayworkers. The dayworkers were treated for hypertension to a greater extent than the shift workers, which could explain the modest BP-change among them. However, we performed a sensitivity analysis after the 3-year follow-up. Here we found that among those who started BP-treatment the therapy outcome was mixed, likely due to nonadherence to the treatment of hypertension. Therefore, it is likely that the 4-year results are in agreement with our results after the 3-year follow-up [20].

The most significant finding following the four years of follow-up was the obvious increase in PWV among shift workers. At baseline registration the shift workers were on average 40 years old with a mean PWV of 7.6 m/s [15] which is exceeding that of reference values which suggests a mean PWV of 7.2 m/s for the similar age group [14]. During the four years of follow-up, the observed mean annual increase in PWV among shift workers was 0.34 m/s. This is more than twice the figures measured in normal population samples based on cross-sectional data [25,26], and three times that of the dayworkers. This is striking since the older and less healthy dayworkers should increase more in PWV than the younger shift workers given that the increase in PWV from the age 50 years or older is steeper than for younger individuals [26,27].

The results of the 11 papers presented in the review of shift work and arterial stiffness were conflicting when it comes to identify increase in PWV, two in favour and two not being able to detect any association [18]. Our results are, however, in line with cross-sectional studies which link shift work including work at night to increased PWV and thus an increased risk for arteriosclerosis [28,29]. The increase in PWV could be explained by many factors. Traditional risk factors known to be associated with shift work [30,31] such as smoking, less physical activity, high BMI, and high cholesterol values are unlikely to explain the association between arterial stiffness and shift work since we found no difference between day and shift workers for these factors in the 3-year follow-up study [20]. Lack of melatonin appearing during “light at night” has been suggested as a causal factor for increased CVD since melatonin has an antioxidant effect, lowers blood pressure, and reduces coagulability [32]. More likely the circadian disruption and sleep restriction result in arterial stiffness through up-regulation of genes involved in inflammation and the humoral immune system and down regulation of genes involved in Th1 immunity, and by decreasing reverse cholesterol transport [33,34], but also by affecting the vessels directly [35]. Furthermore, arterial stiffness could be caused by endothelial dysfunction, and inflammation results in overproduction of abnormal collagen and reduces normal elastin resulting in vascular stiffness [36]. All in all these mechanisms point to a causal link between loss of sleep, inflammation and arterial stiffness [17,20].

The changes in the shift schedule at one of the plants did not affect the PWV results. One explanation being that the reduction of number of night shifts/consecutive night shifts was limited. The new shift schedule yields ≥ 3 consecutive night shifts ≥16 times annually which slightly exceeds the corresponding figure of >15 times resulting in an excess risk for cerebral vascular disease in Bigert’s study [7]. As for the total number of annually worked night shifts, the novel shift schedule assigned the shift worker with more than 50 nights which is in excess of the 30 worked night shifts cut off in Bigert’s study [7].

Studies suggest, on a general basis, that arterial stiffness could be mitigated by sodium restriction, exercise-based intervention, weight reduction, smoking cessation, and BP and statin treatment [10,11,13]. At baseline in the present cohort-study, self-reported PA, with a high degree of intensity, was associated with lower values of PWV [15]. A recent research letter reports favorable improvements in arterial stiffness of PA prior to night shifts [37]. However, an 8-week intervention with supervised high intensity PA three times a week in a sub-group within the present cohort did not result in PWV-mitigation [16], but it seems that a temporary cease in night work reduces PWV [21].

Shift work is associated with a high use of tobacco products [38]. The use of “snus”, being common in the Nordic countries, is in the present study more prevalent among shift workers than among dayworkers, and positively correlated with sleep score. Tobacco use affects sleep quality [39] increasing the risk of insufficient sleep [40] possibly through nicotine stimulation of sleep regulating neurotransmitters such as dopamine and serotonin [41].

A strength of the present study is its prospective design with the assessment of BP and arterial stiffness. On all occasions of data collection during follow-up, we perform the measurements of BP and arterial stiffness shortly after summer vacation and the same technician performed the arterial stiffness measurements. A limitation of the study is the inclusion of new workers. In 2019 and 2020 we recruited 20 more shift workers and in 2022 additionally 29 but also three dayworkers wanted to take part in the study. This makes the present study a mix of a cross sectional and a follow-up study. However, the novel participants will take part in the study the last years, and this will strengthen the quality of the data at the final follow-up. Another limitation to be considered is that the workers are different when it comes to their “circadian time” relative to our measurements since shift workers are involved in night shift work. This could have an impact of the mean results of PWV and BP measurements. However, Drager et al. [42] found no differences between BP and PWV measurements taken in healthy individuals between 8 AM and 8 PM.

Self-selection of fit, highly motivated shift workers who were interested in taking part in the study could pose a problem as more shift workers from plant A than plant B participated at baseline registrations. At the 3-year and 4-year follow-up, however, the participation rate is similar in the two plants. Another study limitation is sleep registration as sleep was reported by questionnaire only at follow-up and no objective registrations of sleep quality are available.

## 5. Conclusions

Arterial stiffness, by the means of PWV, more than doubles as expected during a 4-year follow-up of industrial shift workers. This may increase the risk for future CVD. A small reduction in number of night shifts and consecutive night shifts does not significantly mitigate arterial stiffness.

## Figures and Tables

**Table 1:** Differences in demographics and unadjusted outcome measures in shift workers and dayworkers in 2022.

	Dayworkers		Shift Workers		Differences between Shift vs. Dayworkers in 2022		
Parameter	No	Mean	No	Mean	Diff.	95% CI	*p*-Value
Age (years)	25	47.2	84	39.7	−7.4	−12.8, −2.0	0.008
Women, (No)	2		6				
BMI (kg/m^2^)	25	27.3	84	27.3	−0.09	−1.5, 1.7	0.91
PA (min/week)	25	89.2	81	107.1	17.9	−29.8, 65.6	0.46
Smokers, (No)	1	0.04	15	0.18	0.14	0.02, 0.25	0.02
Pack-years, (No)	25	9.4	84	5.5	−3.9	−9.3, 1.5	0.15
Snuffers, (No)	6	0.24	38	0.47	0.23	0.02, 0.44	0.03
Years as snuffers	24	3.3	81	5.8	2.5	−0.6, 5.6	0.12
Sleep score, (BIS)	25	8.7	82	14.4	5.7	2.4, 8.9	<0.001
Years in shift, (No)	25	5.8	82	12.9	7.1	4.0, 10.2	<0.001
BP medication	9	0.36	9	0.11	−0.25	−0.46, −0.04	0.02
Statin users	4	0.16	6	0.07	−0.09	−0.25, 0.07	0.28
sBP (mmHg)	25	137.1	84	126.0	11.1	3.5, 18.7	0.006
dBP (mmHg)	25	87.8	84	84.2	3.6	−0.1, 7.3	0.06
AP (mmHg)	25	11.4	84	8.7	2.7	−0.7, 6.1	0.12
cPP (mmHg)	25	41.0	84	37.1	3.9	0.7, 7.1	0.02
cSP (mmHg)	25	117.8	84	110.8	7.0	1.4, 12.7	0.02
cDP (mmHg)	25	76.9	84	73.7	3.2	−1.0, 7.3	0.13
PWV (m/s)	24	8.6	84	8.1	0.5	−0.3, 1.4	0.22
RHR (beats/min)	25	65.0	84	70.5	−5.5	−9.8, −1.2	0.01

No, total number of individuals in 2022; *p*-values < 0.05; BMI, body mass index; PA, physical activity of a high degree of intensity, self-reported; sBP, systolic blood pressure; dBP, diastolic blood pressure; AP, augmentation pressure; cPP, central pulse pressure; cSP, central systolic aorta pressure; cDP, central diastolic aorta pressure; PWV, pulse wave velocity; RHR, resting heart rate.

**Table 2:** Selected cardiovascular variables among shift workers and dayworkers over the 4-year follow-up.

	Dayworkers					Shift Workers					Shift vs. Dayworkers				
Outcome	No Obs	No Persons	Annual Change	Lower	Upper	No Obs	No Persons	Annual Change	Lower	Upper	Diff. Change	Lower Change	Upper Change	*p*-Value	*p*-Corr
sBP (mmHg)	86	34	−0.32	−1.62	0.99	136	51	0.10	−1.00	1.20	0.41	−1.33	2.16	0.64	0.82
dBP (mmHg)	86	34	0.00	−0.70	0.70	136	51	0.22	−0.37	0.81	0.22	−0.71	1.15	0.64	0.82
AP (mmHg)	86	34	−0.17	−0.67	0.34	137	51	0.00	−0.42	0.42	0.17	−0.51	0.84	0.62	0.82
cPP (mmHg)	86	34	−0.14	−0.78	0.50	137	51	−0.07	−0.60	0.47	0.08	−0.78	0.93	0.86	0.86
cSP (mmHg)	86	34	−0.16	−1.22	0.90	137	51	0.12	−0.77	1.00	0.28	−1.14	1.69	0.70	0.82
cDP (mmHg)	86	34	−0.03	−0.78	0.72	137	51	0.20	−0.42	0.83	0.23	−0.77	1.22	0.66	0.82
PWV (m/s)	83	34	0.09	−0.05	0.23	135	51	0.34	0.22	0.46	0.25	0.06	0.43	0.0085	0.06

No obs/No persons: total number of measurements and individuals during follow-up. All analysis were adjusted for physical activity with a high degree of intensity (min/week), being a daily smoker, packyears, age at baseline and sex; *p*-values < 0.05; sBP, systolic blood pressure; dBP, diastolic blood pressure; AP, augmentation pressure; cPP, central pulse pressure; cSP, central systolic aorta pressure; cDP, central diastolic aorta pressure; PWV, pulse wave velocity.

## Data Availability

Not applicable.

## Supplementary Materials

The following supporting information can be downloaded at: https://www.mdpi.com/article/10.3390/jcdd10020070/s1, Table S1: Selected cardiovascular variables among shift workers at the two plants over the last year of follow-up; Table S2: Annual change in selected variables in shift workers comparing the new 12-shift schedule with the ordinary 5-shift schedule. All available measurements from both plants are utilised in the analysis.
