# Supplementary material for: The Prolonged Effect of Shift Work and the Impact of Reducing the Number of Nightshifts on Arterial Stiffness—A 4-Year Follow-Up Study
Source: J Cardiovasc Dev Dis. 2023 Feb 6;10(2):70. doi: 10.3390/jcdd10020070 (PMC9961201; doi:10.3390/jcdd10020070)
Supplement: Supplementary file 1 [file jcdd-10-00070-s001.zip › jcdd-2181256-supplementary.pdf]

Supplementary Table S1. Selected cardiovascular variables among shift workers at the two plants over the last year of follow-up.

Outcome	Nobs-Plant A	Npers-Plant A	Change-Plant A	Lower-Plant A	Upper-Plant A	Nobs-Plant B	Npers-Plant B	Change-Plant B	Lower-Plant B	Upper-Plant B	Diff-Change	Lower-Change	Upper-Change	*p*-Value
sBP (mmHg)	62	36	2.97	−9.21	15.16	47	26	8.25	−4.02	20.52	5.28	−0.61	11.16	0.08
dBP (mmHg)	62	36	1.22	−7.93	10.37	47	26	3.00	−6.29	12.30	1.78	−2.64	6.21	0.43
AP (mmHg)	62	36	−1.80	−10.10	6.51	47	26	0.10	−8.38	8.58	1.90	−2.11	5.90	0.35
cPP (mmHg)	62	36	0.06	−8.14	8.26	47	26	2.91	−5.47	11.28	2.85	−1.12	6.82	0.16
cSP (mmHg)	62	36	3.37	−9.63	16.38	47	26	5.10	−8.13	18.33	1.73	−4.56	8.02	0.60
cDP (mmHg)	62	36	3.65	−4.66	11.97	47	26	2.76	−5.66	11.18	−0.89	−4.91	3.13	0.66
PWV (m/s)	61	35	0.03	−0.99	1.05	47	26	0.09	−0.93	1.11	0.06	−0.43	0.55	0.81

Nobs/Npers: total number of measurements and individuals during follow-up. All analyses were adjusted for physical activity with a high degree of intensity (min/week), being a daily smoker, pack-years, age at baseline, and sex; sBP, systolic blood pressure; dBP, diastolic blood pressure; AP, augmentation pressure; cPP, central pulse pressure; cSP, central systolic aorta pressure; cDP, central diastolic aorta pressure; PWV, pulse wave velocity.

Supplementary Table S2. Annual change in selected variables in shift workers comparing the new 12-shift schedule with the ordinary 5-shift schedule. All available measurements from both plants are utilised in the analysis.

Outcome	12-Shift vs. 5-Shift in Annual Change	Lower	Upper	*p*-Value	*p*-Corr
sBP (mm Hg)	5.39	−1.49	12.26	0.12	0.50
dBP (mmHg)	27.5	−0.92	6.43	0.14	0.50
AP (mmHg)	−0.54	−3.14	2.05	0.68	0.82
cPP (mmHg)	1.01	−2.37	4.39	0.56	0.82
cSP (mmHg)	1.10	−4.51	6.71	0.70	0.82
cDP (mmHg)	0.09	−3.86	4.05	0.96	0.96
PWV (m/s)	−0.26	−0.81	0.30	0.37	0.82

sBP, systolic blood pressure; dBP, diastolic blood pressure; AP, augmentation pressure; cPP, central pulse pressure; cSP, central systolic aorta pressure; cDP, central diastolic aorta pressure; PWV, pulse wave velocity.
